# Giant muscle hydatid in lower extremity: a rare case with neurological symptoms as the first manifestation

**DOI:** 10.1186/s12879-023-08616-y

**Published:** 2023-10-02

**Authors:** Qiyu Jia, Shuo Wu, Jian Guo, Abudusalamu Alimujiang, Hao Zheng, Jun Zhang, Yingbo Wang, Zengru Xie, Chuang Ma

**Affiliations:** 1 Department of Trauma Orthopedics, The First Affiliated Hospital of Xinjiang Medical University, Urumqi, Xinjiang China; 2 Nephrology Center, The First Affiliated Hospital of Xinjiang Medical University, Urumqi, Xinjiang China; 3 Department of Microrepair and Reconstruction, The First Affiliated Hospital of Xinjiang Medical University, Urumqi, Xinjiang China; 4 Department of Medical Imaging, The First Affiliated Hospital of Zhejiang Chinese Medical University, Hangzhou, Zhejiang China; 5 College of Chemical Engineering, Xinjiang Normal University, Urumqi, Xinjiang China

**Keywords:** Echinococcus granulosus, Hydatid cyst, Muscle, Nerve symptoms, Case report

## Abstract

**Background:**

Human hydatid disease typically occurs in organs such as the liver and kidney. Primary solitary intramuscular hydatid disease, however, is rare.

**Case Presentation:**

We report a case of a giant muscle hydatid in the lower extremity, with neurological symptoms as the first manifestation. The symptoms specifically manifested as intermittent pain in the right lower extremity and numbness in the sole of the right foot. However, there were no obvious abnormalities detected in electromyography and lumbar MRI. Subsequent ultrasonography and calf MRI showed that the patient had cystic lesions in the calf. The patient was initially diagnosed with a muscle hydatid cyst. Treatment involved complete surgical excision of the lesion, and the diagnosis of a hydatid cyst was confirmed through macroscopic and microscopic histopathological examination after the mass was excised. The patient was given oral albendazole, and no recurrence was observed during the 12 months of follow-up.

**Conclusions:**

This case underscores the need to consider hydatid disease when diagnosing soft tissue masses in muscles, particularly in endemic areas. Patients may initially present with atypical symptoms like peripheral nerve issues.

## Background

Hydatid disease is a zoonotic parasitic infection prevalent in pastoral areas. It can affect any human organ, such as the liver, lungs, kidney, bone, and other tissues. However, their presence in muscle tissues is rare, representing about 0.7-0.9% of cases [1]. Muscle hydatid disease often has no distinct clinical manifestations, and symptoms related to other systems may appear first. Here, we report a case of a giant muscle hydatid in the lower limbs, with neurological symptoms as the initial manifestation. This research adds to our understanding of the disease, broadens its potential presentations, and highlights the need to consider muscle hydatid disease when diagnosing neurological symptoms.

## Case presentation

A 62-year-old retired female nurse from Urumqi, Xinjiang, who had lived in the city since childhood, experienced intermittent pain in her right lower limb and numbness in the sole of her right foot for over ten days. No significant abnormalities were found in electromyography and lumbar MRI. The physical examination revealed that the patient’s right calf was slightly swollen, with noticeable tenderness, but no foreign body was detected; the patient dismissed the issue of calf swelling, attributing it to obesity.

A hematological examination showed that the patient’s liver and kidney function, and blood cell indicators, were within the normal range. Only the erythrocyte sedimentation rate (76.00 mm/h) and C-reactive protein (70.70 mg/L) were outside the normal range. An ultrasound examination revealed a mass approximately 4.5 cm in depth, 20.3 cm in length, and 3.0 cm in width in the patient’s right calf posterior tibial muscle, soleus muscle, and other muscle bundles. The mass had clear boundaries and was divided internally by a hyperechoic light band, with cyst-like structures of varying sizes. The small sac demonstrated good sound permeability and changed in a “honeycomb” shape.

**Fig. 1:**
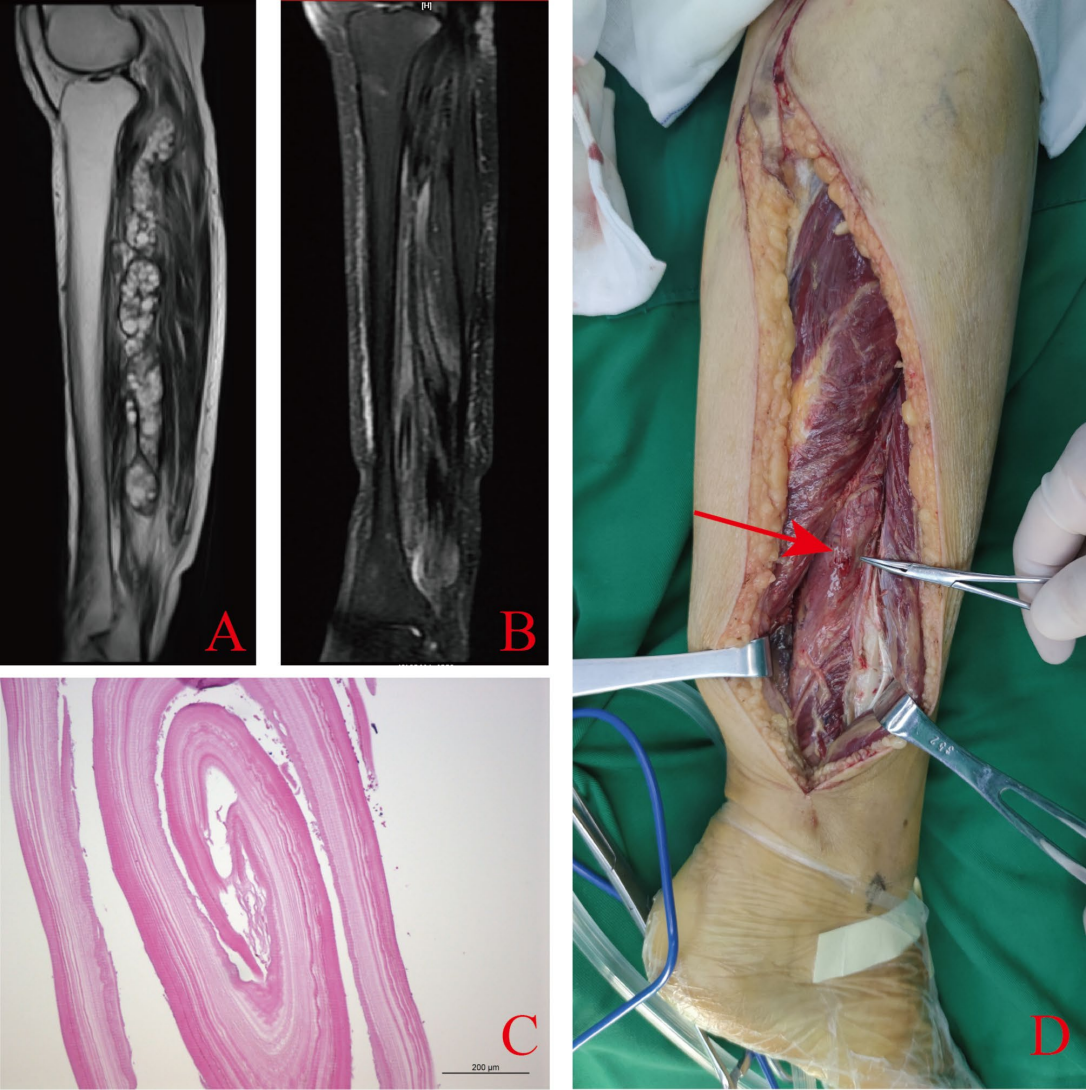
Information regarding the patient’s muscle hydatid cyst. (**A**) MRI of right lower leg at initial presentation. (**B**) MRI of the patient’s right lower leg one year after surgery. (**C**) Pathological section of right calf muscle hydatid cyst. (**D**) Intraoperative picture: muscle hydatid cyst pointed by red arrow

The patient’s calf X-ray showed no evident abnormalities; the right lower limb MRI showed irregular columnar long T1 and long T2 signal changes in the posterior tibialis and soleus muscle spaces behind the right calf. The size was approximately 24.76 cm*3.56 cm*3.82 cm, and it displayed multiple vesicle-like changes. Multiple lines of slightly longer T1 and short T2 signal separation and vascular void signals existed. Slightly high signals were visible in the muscle groups and subcutaneous soft tissues of the upper right calf during the fat suppression sequence (Fig. 1A).

The patient was initially diagnosed with echinococcosis of the right calf and decided on a treatment plan that combined surgery and postoperative medication. The surgical plan involved the gentle separation of the outer capsule, aiming for complete resection while keeping the outer capsule intact to ensure that the capsule fluid does not spread in the surgical area (Fig. 1D). Postoperative microscopic histopathological examination confirmed hydatid cyst (Fig. 1C). Two days post-operation, the patient began resuming bed-based exercises, and two months later, she was able to slowly walk with the aid of crutches. After the operation, the patient began taking albendazole tablets (15 mg/kg) and regularly reviewed liver and kidney function. After two years of follow-up, no recurrence was detected on MRI (Fig. 1B). Additionally, the patient conveyed contentment with the outcome of the treatment.

## Discussion and conclusions

Hydatid disease is a zoonotic disease caused by tapeworms, with the most common type being Echinococcus granulosus. It is primarily found in Mediterranean countries, the Middle East, South America, and East Africa [2, 3]. While hydatid disease can affect any organ in the human body, the occurrence of a primary hydatid cyst in soft tissue and muscle without evidence of disease in the liver or lungs is extremely rare [4, 5]. The effective filtration of the liver and lungs makes it difficult for echinococcal larvae to reach musculoskeletal tissues. Moreover, the high lactic acid content and mechanical factors, such as contraction activities, make muscles unsuitable for the growth of parasites [6].

In cases of soft tissue echinococcosis, a palpable slow-growing mass is the most common clinical finding. The clinical manifestations are caused by compression of the affected organ [7]. However, to the best of our knowledge, there have been no previous reports of hydatid cysts in muscle tissue presenting with peripheral nerve symptoms as the initial manifestation. In the present case, the patient’s main symptom was numbness in the sole of the right foot and right calf. Clinicians often face challenges in diagnosing hydatid cysts in muscle tissue, as the peripheral nerve symptoms can be nonspecific. Furthermore, the patient presented with only mild swelling in the right calf at the initial visit, without any apparent disfigurement. It is important to note that the patient herself disregarded the calf swelling, attributing it to her own body size. Considering that the cyst, despite its large size, was located deep within the calf muscles, it is likely that it did not directly affect the appearance of the calf. This further increased the difficulty of diagnosis. We believe that the numbness in the plantar region of this patient’s foot is primarily due to the presence of a large hydatid cyst located between the posterior tibial muscle and soleus muscle, which compresses the tibial nerve and its branches. The nerves supplying the ankle and foot originate from the calf, and they mainly stem from the lumbosacral nerve plexus, particularly the tibial nerve, peroneal nerve, and saphenous nerve. The tibial nerve is a continuation of the sciatic nerve trunk. In the popliteal fossa, the tibial nerve runs alongside the popliteal artery. In the lower leg, it descends deep within the soleus muscle in tandem with the posterior tibial artery, eventually wrapping around the posterior aspect of the medial malleolus. It further divides into the lateral plantar nerve and the medial plantar nerve. During its descent, the tibial nerve innervates all the posterior muscle groups and supplies the plantar muscles. The tibial nerve gives rise to numerous terminal branches within the ankle canal, and it, along with its branches, plays a crucial role in the development of pain, coldness, and numbness associated with the mid and hindfoot.

Preoperative diagnosis of hydatid disease is critical to avoid cyst rupture and dissemination, leading to recurrence. When evaluating this patient, we considered several potential etiologies based on clinical presentation and imaging findings. Differential diagnoses included soft tissue tumors such as fibrosarcoma which typically demonstrate distinct morphologies [8]. Chronic conditions such as tuberculosis, hematoma, myositis, and muscular infections were also entertained. Given the patient’s residence in an endemic region, echinococcosis remained a plausible possibility, particularly involving the musculature. Radiological evaluation revealed a multilocular cystic structure on ultrasound and MRI, consistent with previously described hydatid lesions containing daughter cysts. Clinically, the lesion was mainly associated with peripheral neurological symptoms, consistent with local effects from direct compression of surrounding tissues by the mass. Taken together, these clinical and radiological features supported echinococcal etiology over other considerations. While hydatid disease rarely involves soft tissues, it is important to include in the differential for musculoskeletal masses in endemic populations [9, 10]. With preoperative diagnosis of hydatid cyst, percutaneous needle biopsy carrying risks of dissemination can be avoided. Based on the collective findings, we established a preliminary diagnosis of primary muscular hydatid cyst prior to surgical excision and histopathological examination.

In general, imaging evaluation is critical for the preoperative diagnosis of hydatid disease. Serology alone is insufficient to diagnose echinococcosis [11]. The diagnosis relies on identifying a hydatid cyst in tissues. Utilizing all available imaging methods significantly contributes to the preoperative diagnosis. Different imaging modalities are complementary and often provide a definitive preoperative diagnosis [7]. Ultrasound and CT imaging can reveal a calcified cyst wall, microcalcifications in daughter cysts, and different fluid densities between the cysts and surrounding organs [7, 12]. MRI is the preferred examination when hydatid disease is suspected. Classic MRI findings include a multivesicular cyst, an intense rim on T2-weighted images, or a detached membrane [13, 14]. The MRI scan demonstrated a multilocular lesion with several daughter cysts inside a mother cyst.

The choice of treatment modalities depends on the cyst’s anatomical location, its relation to major anatomical structures, the number of cysts, the patient’s general health status, and the surgeon’s experience. Surgery is the preferred therapeutic approach for muscular hydatid disease. During the operation, the surgical field should be fully exposed, and the outer capsule should be gently separated for complete resection while keeping the outer capsule intact. Care must be taken to prevent anaphylactic shock caused by cyst fluid leakage. Scar tissue can be removed as well. Additionally, thoroughly rinsing the surrounding soft tissues with hypertonic saline helps prevent recurrence. Apart from anaphylactic shock, complications associated with muscle hydatid cyst primarily involve local dissemination upon rupture [1]. Additionally, the cyst’s presence can exert pressure on blood vessels and nerves, resulting in localized pain and numbness. In cases where the cyst infiltrates surrounding tissues entirely, complete excision may be impeded. In inoperable cases, percutaneous aspiration, infusion of scolicidal agents, and reaspiration (PAIR), guided by imaging (ultrasound or CT), can be used as an alternative to surgery [14, 15]. Postoperative albendazole therapy is typically given for six weeks to reduce the risk of recurrence [16]. Long-term clinical evaluation is necessary after surgery, with regular follow-up scheduled every three months. Patients presenting with neurological symptoms should be closely monitored for their neurological recovery, while ultrasound and MRI/CT scans should be utilized to detect any potential recurrence or metastasis of the primary lesion.

Based on the findings of this case, we emphasize considering hydatid disease in the differential diagnosis of soft tissue masses in muscles, particularly in endemic areas. Patients may seek medical attention with atypical complaints, such as peripheral nerve symptoms.

## Data Availability

The datasets analyzed during the current study are available from the corresponding author on reasonable request.

## Abbreviations

MRI: magnetic resonance imaging
CT: computed tomography
PAIR: percutaneous aspiration, infusion of scolicidal agents, and reaspiration
